# Building a rheumatology biobank for reliable basic/translational research and precision medicine

**DOI:** 10.3389/fmed.2023.1228874

**Published:** 2023-09-07

**Authors:** Elisa Assirelli, Susanna Naldi, Veronica Brusi, Jacopo Ciaffi, Lucia Lisi, Luana Mancarella, Federica Pignatti, Lia Pulsatelli, Cesare Faldini, Francesco Ursini, Simona Neri

**Affiliations:** ^1^Medicine and Rheumatology Unit, IRCCS Istituto Ortopedico Rizzoli, Bologna, Italy; ^2^Laboratory of Immunorheumatology and Tissue Regeneration, IRCCS Istituto Ortopedico Rizzoli, Bologna, Italy; ^3^1st Orthopedic and Traumatology Department, IRCCS Istituto Ortopedico Rizzoli, Bologna, Italy; ^4^Department of Biomedical and Neuromotor Sciences (DIBINEM), Alma Mater Studiorum, University of Bologna, Bologna, Italy

**Keywords:** biobanking and biorepositories, rheumatology, precision medicine, standard operating procedures, quality controls

## Abstract

Research biobanks are non-profit structures that collect, manipulate, store, analyze and distribute systematically organized biological samples and data for research and development purposes. Over the recent years, we have established a biobank, the Rheumatology BioBank (RheumaBank) headed by the Medicine and Rheumatology unit of the IRCCS Istituto Ortopedico Rizzoli (IOR) in Bologna, Italy for the purpose of collecting, processing, storing, and distributing biological samples and associated data obtained from patients suffering from inflammatory joint diseases. RheumaBank is a research biobank, and its main objective is to promote large-scale, high-quality basic, translational, and clinical research studies that can help elucidate pathogenetic mechanisms and improve personalization of treatment choice in patients with rheumatoid arthritis (RA), psoriatic arthritis (PsA) and other spondyloarthritides (SpA).

## 1. Introduction

Research biobanks are public (non-profit) or private institutions that gather, handle, store, analyze, and disseminate systematically organized biological specimens and data for use in research and development projects. The main aim of biobanks over “traditional” sample collections is to ensure the integrity of both biological and associated clinical data, thus supporting a variety of research areas by providing high-quality samples, consistent data, and dedicated expertise. This scientific scope is achieved in the context of a solid quality system and in respect of the participants’ rights and *ad hoc* regulations (1). Indeed, the biological material and related data are processed solely by authorized and adequately trained personnel; access to data storage systems and privileges are controlled by appropriate security measures and total traceability of the sample from collection to storage, is guaranteed by uniform procedures such as best practices (2) and International Organization for Standardization (ISO) standards (namely ISO 9001:2015 and UNI 20387:2018).

Research biobanks usually deal with specific diseases or restricted cohorts of donors/patients (3, 4); the sample stored following biobanking standards ensure reproducibility of scientific results and can be transferred to other researchers (following strict rules to ensure donors privacy) and assembled with other sources to build up large cohorts, in order to meet broad research needs (extending beyond those of the hosting institution) spanning from elucidation of pathogenetic mechanisms, to development and validation of innovative diagnostic tools, discovery of biomarkers for prognostic and therapeutic stratification, and development of novel therapeutics (5). Other advantages include the involvement of stakeholders, patients’ associations (especially regarding ethical and legal aspects), and potential commercial partners. Finally, biobanks represent the basis for the development of national and international networks since standardized processes and procedures are easier to share among structures. Actually, in 2013, biobanks were proposed to be networked by the European Commission with a pan-European project, the Biobanking and BioMolecular Resource Research Infrastructure- European Research Infrastructure Consortium (BBMRI-ERIC) for a transition from individual research tools to complex international research infrastructures. However, this is a complex and challenging process and is still at an early stage (6).

As a result, biobanks are a strategic resource for influencing the long-term path of medical research and a powerful substrate for high throughput OMICS studies and integration with rapidly improving big data handling techniques, including artificial intelligence and deep le (lines)arning methods, to reach the goal of “personalized medicine” for an ever-growing number of human diseases (7). The strategic nature of biobanks is indeed recognized by the European Community in its framework programs encouraging their development in terms of quality, interoperability, and livelihood, with the most recent example in the ninth framework program Horizon Europe 2021–2027 (pillar “Excellent Science”) focused on research quality or the Italian National Recovery and Resilience Plan (PNRR) (8).

On this premises, we established a rheumatology BioBank, led by the Medicine and Rheumatology Unit of the IRCCS Istituto Ortopedico Rizzoli (IOR) in Bologna, Italy (RheumaBank@IOR), with the goal of collecting, processing, storing, and distributing biological samples and associated data obtained from patients with rheumatic diseases. The RheumaBank, along with two other biobanks (Genetic Biobank and Musculoskeletal Tumor Biobank), is part of the coordinating unit Biological Resources Centre (CRB), member of BBMRI and affiliated to a pilot project promoted by the Italian Ministry of Health (MOH) for the accreditation to the UNI 20387:2018 “Biotecnology-Biobanking-General requirement for Biobanking” standard (henceforth referred to as UNI 20387). This participation is the first step toward obtaining UNI 20387 certification, which is critical for recognizing biobank’s operating quality standards (9). Whole blood, serum, urine and isolated peripheral blood mononuclear cells (PBMC) are currently collected in the RheumaBank, after obtaining specific informed consent from patients. RheumaBank donors are patients suffering from rheumatoid arthritis (RA), psoriatic arthritis (PsA) and other axial or peripheral spondyloarthritis (SpA), that cumulatively account for the vast majority of patients with inflammatory joint diseases seen in rheumatology clinics worldwide.

To present, the RheumaBank has already enrolled over 800 donors and stores over 4,200 samples divided into about 14 aliquots for each sample. These numbers include historical collections related to prior research projects carried out at the Medicine and Rheumatology unit, as well as samples newly enrolled in the Biobank from 2022 onwards. Samples are stored in special −80°C ultra freezers and in liquid nitrogen containers located in restricted rooms with limited access. The samples collected and processed by the Rheumatology Biobank are available to the scientific community for use in rheumatology research projects, and their traceability and controlled storage characteristics ensure research with reliable and repeatable results, a prerequisite for the transferability of knowledge to clinical practice.

The aim of this paper is to describe the methodologies used to establish the path for collection, handling, and storage of biological samples in RheumaBank, in accordance with quality standards that ensure traceability and monitoring throughout the process.

## 2. Materials and methods

### 2.1. Patients’ enrollment and informed consent

The RheumaBank enrollment criteria are predefined as follows: (a) adults (≥18 years) with newly diagnosed RA, PsA or SpA (predominantly axial – AxSpA or peripheral – pSpA); (b) adults with a past diagnosis of RA, PsA or SpA who are starting any biological disease modifying anti-rheumatic drugs (bDMARDs) or Janus kinase inhibitors (JAKi). Samples are obtained at the time of first recruitment or in the event of a major therapeutic change (T0) (e.g., switch from a bDMARD to another of the same class or swap to a bDMARD with different mechanism of action or a JAKi) and then after 3, 6, and 12 months. Subsequently, if treatment remains stable, data are collected every 12 months.

As a general rule, biobanks have a very stringent policy on ethical issues, privacy, and personal data protection. The collection of data and samples takes place only after proper information has been delivered to the patient and written consent has been obtained. Particularly important to the procedure and a prerequisite for the expression of free and fully informed consent is the information phase (10). Correctly informing the patient about participating in a biobank entails more than simply presenting the risks, advantages, and potential alternatives; it constitutes a time of care (11) and calls for starting a bilateral discussion, responding to the patient’s inquiries, and confirming that the patient has understood what has been explained. Only at this stage, the consent form is administered. The form was developed in collaboration with a medical legal consultant and is complemented by an information sheet recapitulating the aim of research biobanks and the benefits and drawbacks of patient’s participation, covering both data collection and specimen use. It is successively submitted at each time point.

The consent information sheet is structured in 18 paragraphs that provide answers to the questions listed in Panel A:

**Panel A:** List of the questions addressed by the consent information sheet distributed to donors prior to sample recruitment.

What is a research biobank? What activities are carried out by a research biobank? Can samples stored in the biobank be used in other research areas? Am I obliged to participate? What advantages may I anticipate from participating? What are the potential risks of participating? How and how long will my samples be stored? How will my biological material and personal data be handled? Will genetic studies be conducted on my biological material? What procedures will be adopted for the processing of my personal data? How will my genetic data be handled? To whom could my data be disclosed? Could my biological samples be transferred? What methods does the biobank employ to determine which research initiatives ought to be given the preserved samples? What potential applications can the findings of the research made possible by the samples preserved in the biobank have? Will my samples be eligible for patent development? Who can I contact for further information? Will it be possible to cancel my participation agreement?

The patient’s signed consent form, obtained during each individual visit, is stored in a locked cabinet located in a room with restricted access. The ethical and regulatory framework under which the consent form was built is reported in Supplementary material S1. The complete informed consent in Italian language is available online at https://www.ior.it/centro-risorse-biologiche.

### 2.2. Data collection

Clinical data of patients enrolled are acquired following each visit and stored in a dedicated database built using Research Electronic Data Capture (REDCap) electronic data capture tools (12, 13). REDCap is a secure, web-based software platform designed to support data capture for research studies, providing (1) an intuitive interface for validated data capture; (2) audit trails for tracking data manipulation and export procedures; (3) automated export procedures for seamless data downloads to common statistical packages; and (4) procedures for data integration and interoperability with external sources.

Data collected include date of birth, sex, menopausal state, smoking status, familial history (details are provided as Supplementary material S2), past medical history (details are provided as Supplementary material S2), anthropometric measurements (weight, height, waist circumference), disease and symptoms duration, clinical features, clinimetric measures, current and past treatments, laboratory markers. Clinical features include: fulfillment of classification criteria for RA (14), PsA (15), AxSpA (16) or pSpA (17), presence of extra-articular manifestations (RA, PsA, SpA) or erosive disease (RA), presence and duration of cutaneous or nail psoriasis (PsA, SpA), previously recognized (before T0) enthesitis, dactylitis, or axial disease (PsA, SpA), history of inflammatory bowel disease or uveitis (PsA, SpA). Disease-specific clinimetric measures and laboratory markers collected are reported in Table 1. Finally, information on dosing, duration, and reason for discontinuation are recorded for all current and previous medications of rheumatologic interest (e.g., DMARDs, bDMARDs, JAKi, corticosteroids, nonsteroidal anti-inflammatory drugs, paracetamol, opioids); other medications taken during each visit are annotated.

**Table 1:** Serum markers and clinimetric measures recorded at each individual visit for the diseases indicated in the left column.

Serum markers		
RA, PsA, SpA	ESR (mm/h)	Erytrocyte sedimentation rate
RA, PsA, SpA	CRP (mg/dL)	C-reactive protein
RA, PsA	RF (titer)	Rheumatoid factor (IgM)
RA	ACPA (titer)	Anti-citrullinated protein antibodies
PsA, SpA	HLA-B27 (presence)	Human leukocyte antigen B27
Clinimetric measures		
RA, PsA	DAS28	Disease Activity Score including 28 joints (18)
RA	CDAI	Clinical Disease Activity Index (19)
RA	SDAI	Simplified Disease Activity Index (20)
RA, PsA, SpA	HAQ-DI total score	Health Assessment Questionnaire Disability Index (21)
PsA	DAPSA	Disease Activity in Psoriatic Arthritis (22)
PsA, SpA	LEI	Leeds Enthesitis Index (23)
PsA, SpA	ASDAS	Ankylosing Spondylitis Disease Activity Score (24)
PsA, SpA	BASDAI	Bath Ankylosing Spondylitis Disease Activity Index (25)
PsA, SpA	# Dactylitis	Number of digits with dactylitis (26)

PsA, psoriatic arthritis; RA, rheumatoid arthritis; SpA, spondyloarthritis.

### 2.3. Sample collection

Standardization of operating procedures directly determines the sample quality and its ultimate field of use. After obtaining consent, biological samples are collected according to the scheme shown in Table 2. Standard operating procedures for samples procurement, processing and storage are based on the best practices recommended by the International Society for Biological and Environmental Repositories (2), the NCI Best Practices for Biospecimen Resources (27) and the standard ISO 9001:2015 and UNI 20387:2018.

**Table 2:** Samples collection schedule.

Timepoint	Baseline (T0)	Month 3 (T3)	Month 6 (T6)	Month 12 (T12)	[…]*
Whole blood (clot accelerator with gel separator tube)	✓	✓	✓	✓	✓
Whole blood (EDTA tube)	✓		✓	✓	✓
Urine (no preservatives)	✓				

* After the first 12 months, data are collected at 12-month intervals until lost from follow-up.

EDTA, ethylenediaminetetraacetic acid.

Blood and urine are collected from participants into vacutainer tubes according to the schedule reported in Table 2. The hypodermic needle is attached to these vacutainer tubes during venipuncture, and the tiny internal suction pulls enough blood to fill each tube with the necessary additives. The participant’s urine is transferred from the urine collecting vessel into a vacutainer using a similar technique. The following information is noted: time of sample collection, time of sample pick up by the driver who transport them to the biobank, and arrival time at the biobank. One and a half hours is the maximum interval time between sample collection and arrival at the biobank. Meanwhile, samples are stored at +4°C. During transport to the biobank, the material is kept at room temperature (15′ average transport time).

### 2.4. Sample processing

**Figure 1:**
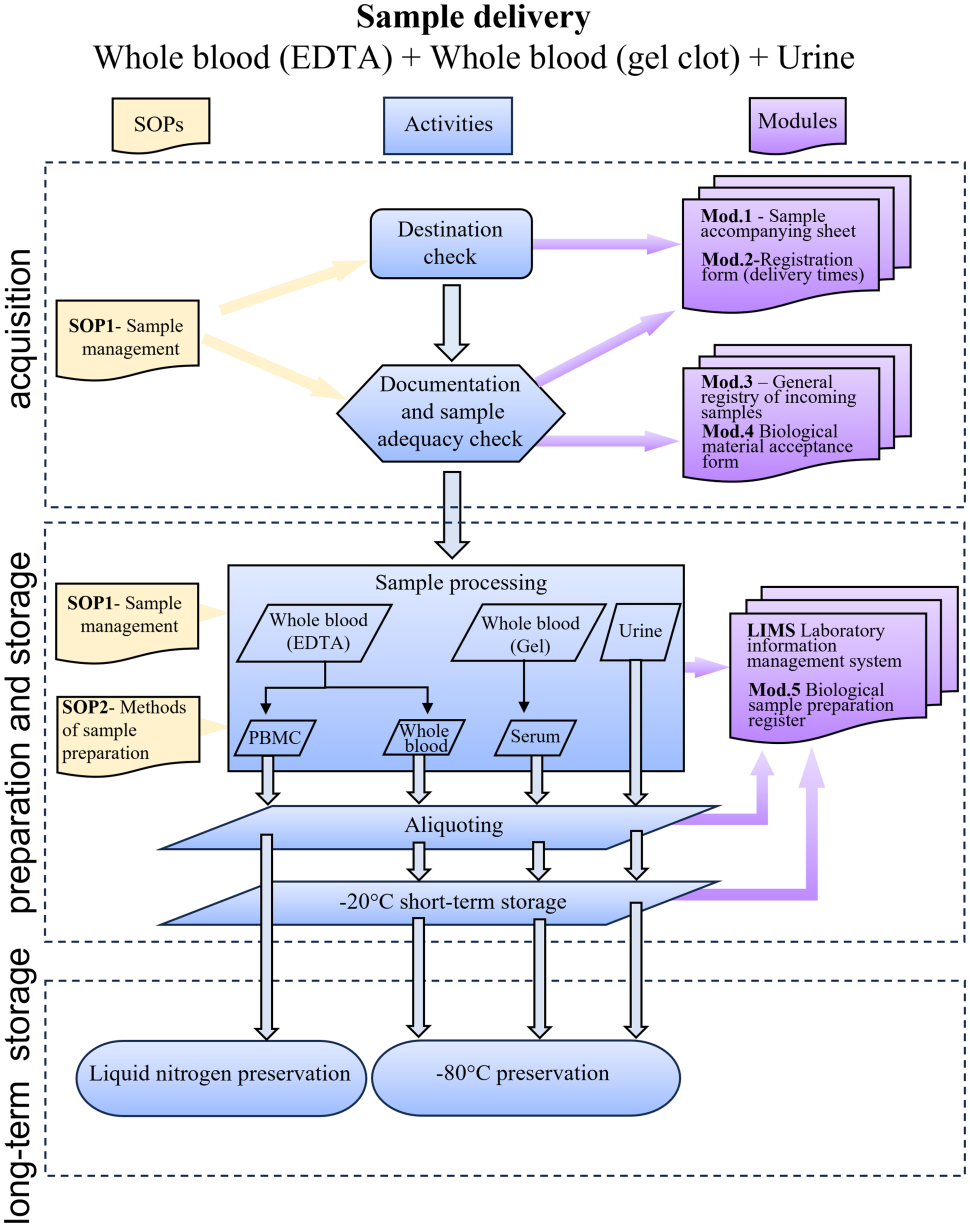
Flow chart showing the RheumaBank workflow from sample delivery to storage. SOP, standard operating procedures; Mod, module; PBMC, peripheral blood mononuclear cells; LIMS, Laboratory Information Management System.

Figure 1 summarizes the sample’s path from the time it arrives at the biobank until it is finally stored.

The samples are delivered with an accompanying sheet that includes patient’s name, birthplace, date of birth, sex, eligibility criteria, the name of the clinical unit sending the samples, the person who collected the samples, the time and date of the sample collection, the type of samples, the number of tubes, the sending date and the sender’s name (see Supplementary material S3).

The biobank staff records the arrival of the sample, noting the date and time of arrival, and verifies the existence and accuracy of all the information on the accompanying sheet. Each biological sample is given a unique identification code as soon as it is accepted into the biobank. The sample is identified by this code at every subsequent stage of processing, storing, and registration. All information is recorded in the RheumaBank biological sample preparation register (see Supplementary material S4). The association between name and code is stored in a notebook that holds the accompanying sheets, in a confined and closed environment, reserved to the authorized biobank staff. Non-compliant samples or documentation are also registered. Non-compliant samples are samples lacking one of the following essential information: time and date of sampling, information about presence of informed consent from the patient or parental authority, sample identification. Also, if the number of containers arrived is different from the number of containers sent. The absence of sample identification is the only non-compliance preventing acceptance. All other infractions are regarded as minor and are dealt with in accordance with the biobank’s regular operating procedures.

### 2.5. Preparation of aliquots

All the biological material is aliquoted as indicated in Table 3. Aliquots are directly prepared from non-coagulated blood (blood in EDTA), urine and synovial fluid without further manipulation, whereas serum and peripheral blood mononuclear cell (PBMC) preparation requires the application of specific protocols.

**Table 3:** Number and type of biological sample aliquots obtained from each donor.

Vacutainer tube	Fraction	Number of aliquots	
		−80°C	Liquid nitrogen
EDTA (5 mL)	Whole blood	2	
	DMSO PBMC		2–3
Serum separation tube (5 mL)	Serum	7	
Urine	Urine	3	

DMSO, dimethyl sulfoxide; EDTA, ethylenediaminetetraacetic acid; PBMC, peripheral blood mononuclear cells.

#### 2.5.1. Serum

Different centrifugation techniques affect the serum’s composition and quality, which may change the samples’ biochemical signature. Centrifugation techniques must be thoroughly standardized to guarantee sample comparability. Choosing the centrifugation time and speed typically involves considering the serum’s intended use. The centrifugation duration and speed are, however, predetermined in biobanks based on scientific literature to cover the bulk of any conceivable application because samples are made available for future analyses (28). Blood sample quality is mostly affected by hemolysis as it impacts the accuracy of many laboratory tests, particularly chemistry assays (29). If hemolysis is visually noticed either before or after centrifugation, this information is recorded in the “notes” area of the RheumaBank biological sample preparation register (30–32). The serum is aspirated and aliquoted into labeled storage tubes (at least 150 μL/tube) after being centrifuged in the serum separation vacutainer (BD Vacutainer^®^, Becton, Dickinson)^1^ at 1500 × *g* for 15′ at 4°C in a refrigerated centrifuge following manufacturer instructions, and it is then stored at −80°. Aliquoting is fundamental to minimize resampling or freeze–thaw events, both of which have been shown to affect downstream analysis (33). The choice of aliquot volume was made trying to have as much versatility as possible and adequate suitability for the most common applications (such as enzyme-linked immunosorbent assay (ELISA) tests). On the other hand, the ability to combine more aliquots from the same sample enables the performance of tests that require more material. Researchers requesting samples from the biobank are aware of timelines and methods, allowing them to evaluate whether the samples are suitable for their application purposes.

#### 2.5.2. PBMC isolation

After preparing the whole blood aliquots (200 μl) from the EDTA tubes, the remaining blood is used for PBMC isolation following a standardized protocol carried out under sterile conditions (34). The protocol is described in Panel B. PBMC are frozen in liquid nitrogen after isolation.

**Panel B:** Standardized protocol for PBMC isolation from whole blood.

Before use, preheat the separation density gradient medium at room temperature Prepare the samples at room temperature by diluting the whole EDTA blood 1:2 in Phosphate Buffered Saline (PBS) solution Add the separation density gradient medium (1:3 ratio with diluted blood) to a clean centrifuge tube Carefully layer the diluted blood onto the separation density gradient, avoiding mixing the two phases Centrifuge the tubes at 800 g x 20 min at room temperature Using a sterile pipette, discard the upper layer containing plasma and platelets, thus leaving the mononuclear cell layer undisturbed at the interface Transfer the mononuclear cell layer to a new centrifuge tube Add PBS to a volume of 15 mL and centrifuge the tubes at 500 × *g* for 10 min at room temperature. Repeat this step. The number of PBMC recovered after separation is calculated by counting the cells using a Neubauer chamber. Several aliquots are prepared based on cell number, at least 2 × 10^6^ per aliquot are required for storage, with a maximum of 3 aliquots per sample.

### 2.6. Sample preservation and conservation

Before the final storage at −80°C, serum, urine, and blood samples are maintained at −20°C for a maximum of 2 weeks. PBMC are resuspended in fetal calf serum (FCS) containing 10% dimethyl sulfoxide (DMSO) and preserved at −80°C one night before being transferred to liquid nitrogen for long-term storage. Date and time of sample transfer are recorded in the RheumaBank biological sample preparation register. At the end of the procedure, sample position in the final storage container is recorded in the Laboratory Information Management System (LIMS), along with all the information gathered regarding sample processing (such as timing, number of aliquots, and consent preferences).

Quality controls (QCs) are regularly performed to check the quality of samples and data. QCs are typically run on PBMC and data. We chose against examining whole blood samples because they are primarily used for DNA extraction, whose quality is not considerably affected by the process of long-term storage and thawing (35, 36). For analytes other than DNA, there is growing evidence that, with the exception of a few well-defined classes of (small) molecules, there is little difference between stability in plasma/serum and stability in whole blood (37). This is true even though analytes being measured in plasma or serum will be stored in whole blood for a while during the sampling process and information about their stability during this period would be needed. Therefore, if plasma/serum stability has been demonstrated under the same storage conditions, whole blood stability testing may not always be strictly essential (38). Being impossible to check every single aliquot quality, due to the limited sample amount and the need for economic sustainability, QCs are regularly performed and are based on aliquot selection. To prevent the risk of running out of valuable material, 5 samples for PBMC viability testing are randomly chosen once a year from those for which more than one aliquot of material could be frozen. Additionally, 10 samples are randomly selected for data quality check.

#### 2.6.1. Biological samples QCs

##### 2.6.1.1. PBMC viability test

This test is performed after a freeze/thaw cycle, as described in the following protocol:

1. As soon as the sample has been removed from the liquid nitrogen, thaw it in a water thermostatic bath at 37°C, then dilute it in 10 mL of Roswell Park Memorial Institute (RPMI 1640) medium with 10% FCS
2. Centrifuge the tube at 500 *g* × 5′ at room temperature
3. Resuspend the pellet in 3 mL PBS and count the cells in a Neubauer chamber with eosin dye.
4. Estimate the percentage of living cells and the proportion of recovered cells compared to the amount originally frozen.

After the quality controls are performed, the remaining sample is discarded.

##### 2.6.1.2. Serum sample quality test

From a practical standpoint, it is not feasible to develop protocols to assess the stability of each biomolecule within the sample. As previously described, samples are prepared according to protocols that optimize the overall stability of biomolecules under certain environmental conditions (39). In general, if a particular biomolecule is of interest for the type of sample being stored, it is important to perform analyses to confirm that the storage and handling conditions used allow for accurate determinations of that biomolecule. Regarding our serum samples, when the serum processing protocol was established, tests were performed to check the stability of a panel of biomolecules. Seven molecules (YKL40, MIP-3beta, Il-6, Resistin, MMP-3, EGF, VCAM-1) from a previously published multi-biomarker disease activity (MBDA) score to assess disease activity in RA (40) were chosen for this purpose and measured using a Bioplex multiplexing platform (Biorad) with a Lumiinex assay.^2^ We then evaluated the coefficient of variation (CV) using a cutoff of 20% as quality indicator between two aliquots of the same sample, one tested immediately after cryopreservation and the other after 1 year at −80°C.

##### 2.6.1.3. Data QCs

Ten samples are randomly extracted from the LIMS system. Each sample is checked for missing data as well as consistency between electronically recorded data and data reported on paper forms. All non-compliances are registered.

### 2.7. Sample distribution

Sample distribution is performed following a written procedure, including addresses and links to contact the biobank personnel. Sample distribution will only be considered for research studies that have received approval from an independent Ethics Committee. After receiving the sample distribution request, an institutional biobank review board (composed of three senior scientists including an expert in the field of rheumatology) evaluates whether the study aims match with those of the biobank, to protect depositors’ rights. After the principal investigator and the biobank have signed a material transfer agreement (the “Rheumabank Material Transfer Agreement”), covering samples and data use (for nonprofit research use only), samples are delivered with an accompanying sheet and the requested dataset.

### 2.8. Recovery plan

Biobaking involves the collection of valuable and frequently irreplaceable samples. Sample security must be guaranteed because the loss of even one sample would be a damaging event for the depositor, the research, and the organization hosting the biobank. In addition, the biobank is a costly investment in economic terms for the institution hosting it. Therefore, in case of unforeseen events that could harm facilities and samples, it is critical to prepare risk management, mitigation, and recovery plans. As recommended by the ISO9001:2015 standard, we have created a proactive risk analysis that is updated yearly and takes into account all mitigation strategies and preventive/corrective actions. All the general and operational procedures of the research institution (IRCCS Istituto Ortopedico Rizzoli) in which it is hosted (e.g., monitoring of power generators, filling of nitrogen tanks, custodial procedures) applies to the biobank. In particular, the CRB of our Institution have two backup −80°C empty freezers available and ready for fast biological material transfer in the event of a malfunction or breakage. The −80°C freezers are continuously monitored, and any ongoing adverse event is instantly reported to the biobank staff. An on-call service for technical problems is available 24/7.

## 3. Results

In 2022, a total of 246 patients were enrolled in the RheumaBank. Four different sample types were collected from each patient: whole blood (2 aliquots), serum (7 aliquots), urine (3 aliquots) and PBMC (2 aliquots) for a total of 3444 aliquots at the T0 timepoint, as represented in Table 4. Recruitment for T3, T6, and T12 is ongoing. The number of patients enrolled for each rheumatic disease reflects the prevalence of the condition in the general population, with RA having the highest prevalence (Table 4).

**Table 4:** Rheumatology biobank deposits in 2022, divided according to the rheumatic disease.

Disease	RA	SpA	PsA	Controls	Total
Patients (n)	114	36	59	37	246
Sample type	Number of aliquots				
Whole blood (n)	228	72	118	74	492
Serum (n)	798	252	413	259	1722
Urine (n)	342	108	177	111	738
PBMC (n)	228	72	118	74	492
Total (n)	1596	504	826	518	3444

The different sample types and the number of aliquots for each sample type are indicated. PBMC, peripheral blood mononuclear cells; PsA, psoriatic arthritis; RA, rheumatoid arthritis; SpA, spondyloarthritis.

More than 80% of serum, blood and urine samples were processed within 4 h from collection, and PBMC samples were prepared and stored within 48 h from blood collection in about 90% of samples.

**Figure 2:**
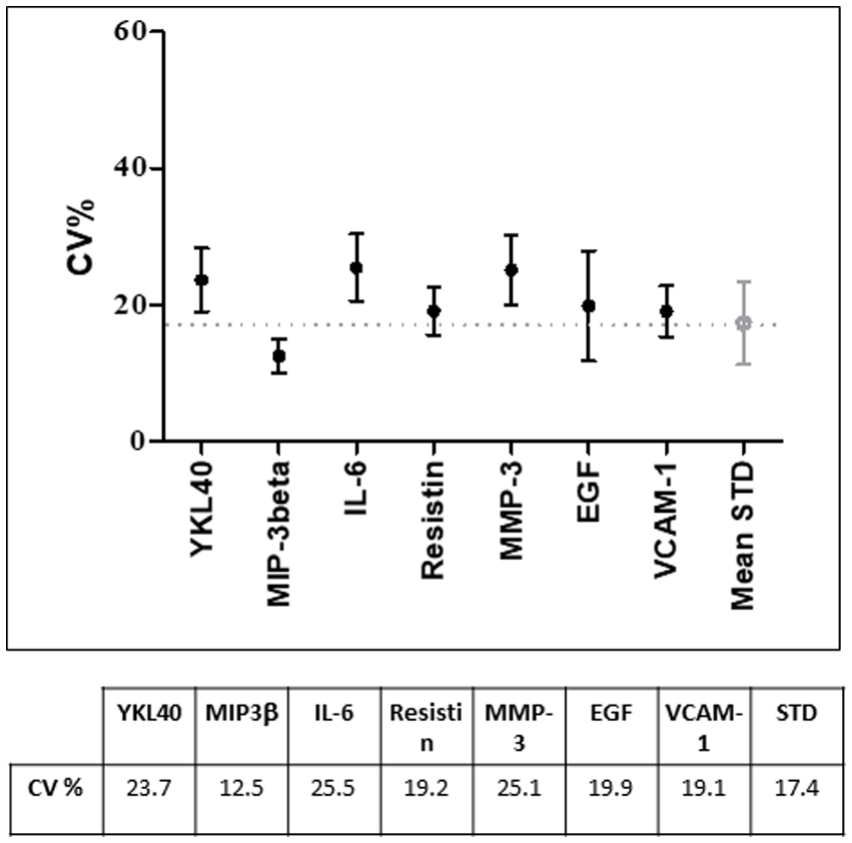
Coefficient of variation (CV%) of the selected biomarkers used for quality control tests on 20 randomly selected serum samples. Under the graph, CV% values are indicated. STD, standard.

The quality tests performed on serum samples showed a coefficient of variation (CV) under the standard medium CV (20%) as shown in Figure 2.

## 4. Discussion

A better understanding of human diseases is strictly dependent on depicting the pathophysiological processes and recognizing the relationship between genotype and phenotype, which, in turn, could lead to the successful development of novel medications and more effective personalization of available treatments. Biobanks play a critical role in ensuring sampling homogeneity and reliability through the implementation of best practices, as well as providing access to a large number of biological samples, thereby accelerating research and allowing for the rapid translation of results into clinical practice (41). In this context, the United Kingdom (UK) biobank is such an excellent example, collecting genotype of about 500,000 individuals associated with extensive phenotype (lifestyle, biomarkers, imaging) data. The UK biobank has already promoted the collection of multiple data sets on the relationship between phenotype and pathogenic genotype, significantly improving our understanding of human functioning in health and disease, confirming the enormous value of this research infrastructure (42).

Rheumatic diseases are common in the general population, yet despite the availability of novel therapeutic options, a significant proportion of patients continue to fail to respond effectively to first-line treatments, pointing to one of the field’s biggest unmet needs (43). Given the heterogeneity of arthritis pathogenesis, the goal of therapeutic strategies should ideally be to target the pathogenic mechanism prevailing in each individual patient. Further, some individuals may have diverse processes concurring to the disease phenotype, resulting in a partial or complete response to a narrowly targeted therapy (44). These factors have resulted in an urgent need for a better understanding of the pathophysiology of these diseases, as well as the identification of biomarkers that can reliably predict the best treatment for a given patient and provide a theoretical basis for the development of novel therapeutics.

In this manuscript we describe the main activities carried at our Institution, a specialistic musculoskeletal research hospital, to build a rheumatology biobank. To the best of our knowledge, RheumaBank is the first biobank in Italy specifically conceived to provide support to basic, clinical, and translational studies in rheumatology, aiming at shade light on the pathophysiological mechanisms and stimulating the development of novel therapeutic molecules in a personalized medicine framework. However, to set up a biobank is a complex task, that requires taking in account several aspects, from those concerning governance and ethics to pure methodological, operational, and technical ones (45).

The first important point to consider is to build an informed consent form taking into consideration all ethical, legal, and social issues while adhering to national and international standards. In our case, we prepared this document in compliance with the guidelines of the Ethical, Legal and Social Issues (ELSI) working group of BBMRI.it, the national node of BBMRI-ERIC, the European research infrastructure for biobanking and biomolecular resources (46). All the preparatory information necessary to sign the consent is provided to patients by healthcare professionals skilled in the field of biobanking; additionally, to encourage patient compliance, blood sampling is scheduled to harmonize with routine health care appointments (47).

Recent articles have emphasized the importance of the reproducibility of scientific data, which is closely linked to the quality of the biological samples (48). Published studies have been discovered to be difficult or impossible to replicate or reproduce, a methodological crisis known as the “replication crisis” (also known as the replicability crisis or reproducibility crisis) (49). Even though the outcomes of preclinical research cannot be replicated, many secondary papers are nonetheless created (50). To counteract this phenomenon, research biobanks use a predetermined procedure for collecting, handling, and storing biological samples that ensures the standardization of the methods, specific training of personnel, and continuous checks on the path and the samples (51, 52). Furthermore, biobanks implement quality controls (on both banked samples and collected data) throughout banking procedures to verify the quality of the samples and, consequently, the reliability of scientific results and their application (53). While the quality controls that must be carried out on cells and nucleic acid samples are well described in the reference CEN standards (54, 55), the publication of a specific standard for serum and plasma samples is still pending.

Our workflow is designed to guarantee sample processing within 4 h after collection. In most applications, this meets the requirements for research use. In cases where stricter conditions are mandatory, such as serum samples used for metabolomics analysis where processing within 90 min is indicated (56), it will be possible to choose the idoneous samples since detailed processing times are always recorded. In this way, those who will have to use biobank samples for research purposes will always be aware of when they were processed and be able to decide which analyses can or cannot be performed (57). Accordingly, we run *ad hoc* experiments to evaluate the stability of specific molecules relevant to the diseases collected in the biobank (58).

All the standard operating procedures developed and described here have been periodically evaluated and improved. The whole process complies with the ISO 9001:2015 standards and an upgrade to the UNI 20387 standard is underway. It should be noted, however, that the standardization and improvement of pre-analytical procedures for *in vitro* studies is an ongoing process. The most up-to-date high priority pre-analytical CEN and ISO standard documents, as well as the corresponding External Quality Assessment (EQA) schemes and implementation tools were the basis for the establishment of our pathway. Not only following adequate standard operating procedures (SOPs) is essential to guarantee research results, but also having qualified personnel, aware of the role and potential of the biobank (59, 60). To reach this goal, a continuous training plan has been foreseen. In particular, the operative biobank personnel attended several training courses on UNI 20387, organized by Accredia, the sole national accreditation body appointed by the Italian government in compliance with the application of the European Regulation 765/2008, and allowed to certificate the compliance to UNI 20387. Furthermore, every member of the RheumaBank staff obtained the certificate to conduct internal audits for the assessment of compliance with the UNI 20387 standard.

Finally, the working environment is undeniably one of the most important aspects of biobank success, requiring the strict collaboration of all involved personnel, beginning with medical staff charged with selecting donors and collecting fundamental clinical data; to healthcare personnel charged with acquiring informed consent, to nursing staff charged with collecting biological samples, to research and technical personnel charged with sample manipulation and storage. The complex biobank workflow will succeed only if all of these components work together to achieve the same aim.

## Data availability statement

The raw data supporting the conclusions of this article will be made available by the authors, without undue reservation.

## Ethics statement

The studies involving humans were approved by Comitato Etico di Area Vasta Emilia Centro della Regione Emilia-Romagna (CE-AVEC). The studies were conducted in accordance with the local legislation and institutional requirements. The participants provided their written informed consent to participate in this study.

## Author contributions

EA, SNa, FU, and SNe: conceptualization and methodology. EA, SNa, and SNe: investigation and data curation. FU: resources and funding acquisition. EA and SNe: writing—original draft preparation. EA, SNa, VB, JC, LL, LM, FP, LP, CF, FU, and SNe: writing, review, and editing. EA, SNe, and FU: project administration. All author has been involved either in the conception and design of the study, or in the acquisition of data, or in the analysis and interpretation of data, or in drafting the article or in revising it critically for important intellectual content, or in the final approval of the submitted version.

## Funding

This study was funded by the IRCCS Istituto Ortopedico Rizzoli, Bologna–Italy (“Ricerca corrente” fund).

## Conflict of interest

The authors declare that the research was conducted in the absence of any commercial or financial relationships that could be construed as a potential conflict of interest.

## Publisher’s note

All claims expressed in this article are solely those of the authors and do not necessarily represent those of their affiliated organizations, or those of the publisher, the editors and the reviewers. Any product that may be evaluated in this article, or claim that may be made by its manufacturer, is not guaranteed or endorsed by the publisher.

## Footnotes

^1^http://www.bd.com/

^2^https://www.rndsystems.com/promotions/luminex-assay-users-guide
