# Supplementary material for: Building a rheumatology biobank for reliable basic/translational research and precision medicine
Source: Front Med (Lausanne). 2023 Sep 7;10:1228874. doi: 10.3389/fmed.2023.1228874 (PMC10513757; doi:10.3389/fmed.2023.1228874)

Supplementary Material

Building a Rheumatology biobank for reliable basic/translational research and precision medicine

Elisa Assirelli^1^, Susanna Naldi^1^, Veronica Brusi^1^, Jacopo Ciaffi^1^, Lucia Lisi^1^, Luana Mancarella^1^, Federica Pignatti^1^, Lia Pulsatelli^2^, Cesare Faldini^3,4^, Francesco Ursini^1,4*^ and Simona Neri^1^

^1^ Medicine and Rheumatology Unit, IRCCS Istituto Ortopedico Rizzoli, Bologna, Italy

^2^ Laboratory of Immunorheumatology and Tissue Regeneration, IRCCS Istituto Ortopedico Rizzoli, Bologna, Italy

^3^1st Orthopedic and Traumatology Department, IRCCS Istituto Ortopedico Rizzoli, Bologna, Italy

^4^ Department of Biomedical and Neuromotor Sciences (DIBINEM), Alma Mater Studiorum University of Bologna, Bologna, Italy

**Correspondence:**

Francesco Ursini: francesco.ursini@ior.it

# Supplementary Figures and Tables

**Supplementary material S1**. Regulatory and ethical frameworks of informed consent.

| **Level** | **Body** | **Document** |
| --- | --- | --- |
| **National** | Italian Government | - Italian Criminal Code, article 50 (1930) - Italian Civil Code, article 5 (1942) - Constitution of the Italian Republic, articles 2, 13, and 32 (1947) - Italian Code of Industrial Property, section IV-bis “Biotechnological Inventions”, articles 81bis and following (2005) |
|  | National Committee of Bioethics and National Committee for Biosecurity, Biotechnologies and Life Sciences | - Guidelines for the Certification of Biobanks (2006) - Biological Sampling for Research Purposes: consensus (2009) - Opinion “Pediatric Biobanks” (2014) |
|  | Italian Data Protection Authority (DPA) | - Resolution n° 146 – Sensitive data (2019) - General authorization No. 8/2014 for the Processing of Genetic Data (2014) - General authorization to Process Personal Data for Scientific Research Purposes (2014) |
|  | Italian Society of Human Genetics | - Guidelines for genetic biobanks (2003) |
| **European** | European Convention on Human Rights | - Charter of Fundamental Rights of the European Union (2000) - Oviedo Convention for the Protection of Human Rights and the Dignity of the Human Being with regard to the Applications of Biology and Medicine (1997) |
|  | European Parliament | - Regulation N°679 – “General Data Protection Regulation”, (2016) |
|  | Organization of European Cancer Institutes (OECI) | - From biobank to research repository: ethical and legal recommendations (2010) |
| **International** | UNESCO | - Universal Declaration on Human Genome and Human Rights (1997) - Universal Declaration on Bioethics and Human Rights (2005) - Universal Declaration on the Human Genome and Human Rights (1997) |
|  | World Medical Association | - Declaration of Helsinki: Ethical Principles for Medical Research Involving Human Subjects (1964, last revised 2008) |

**Supplementary material S2.** Demographics, familial and past medical history information collected.

| **Demographics (all patients)**   - Date of birth (DOB) - Gender - Menopause - Current/past smoker |
| --- |
| **Familial history (all patients)**:   - Rheumatoid arthritis (RA) - Psoriatic arthritis (PsA) - Peripheral spondyloarthritis (pSpA) - Axial spondyloarthritis (AxSpA) - Other systemic autoimmune diseases - Coronary artery disease (CAD) before 60 years of age - Fragility fractures - Inflammatory bowel disease (IBD) |
| **Comorbidities (all patients)**:   - Diabetes (type 1 or 2) - Hypertension - Acute myocardial infarction (AMI) - Stroke/transient ischemic attack (TIA) - Heart failure (HF) - Atrial fibrillation (AF) - Chronic obstructive pulmonary disease (COPD) - Chronic kidney disease (CKD) - Malignancy - Osteoporosis - Fragility fracture - Psoriasis - Inflammatory bowel disease (IBD) - Fibromyalgia - Hip arthroplasty (THA) - Knee arthroplasty (THA) - Shoulder arthroplasty (TSA) - Other disease-related surgical procedures |
| **Rheumatoid arthritis clinical data (individual visit):**  Diagnosis date*  Symptoms onset date*  Extra-articular features experienced to date:   - Rheumatoid nodules - Rheumatoid vasculitis - Sicca syndrome - Interstitial lung disease   Erosive disease  Current RA treatment (including dosing and duration)  Past RA treatments (including dosing and duration)  Concurrent medications  Weight (kg)  Height (cm)  Waist circumference (cm)  Erythrocyte sedimentation rate (ESR)  C-reactive protein (CRP)  Rheumatoid factor (titre)  Anti-citrullinated protein antibodies (titre)  Tender joint count (28 joints)  Swollen joint count (28 joints)  Global health (GH)  Disease activity score including 28 joints (DAS-28)  Patient global assessment (pGA)  Physician global assessment (PhGA)  Clinical Disease Activity Index (CDA)  Simplified Disease Activity Index (SDAI)  Health Assessment Questionnaire (HAQ)  * Collected only at baseline |
| **Psoriatic arthritis clinical data (individual visit):**  Diagnosis date*  Symptoms onset date*  Clinical features experienced to date:   - Familial history of psoriasis - Skin psoriasis - Nail psoriasis - Synovitis - Enthesitis - Dactylitis - Axial disease - Uveitis - Inflammatory bowel disease   Current PsA treatment (including dosing and duration)  Past PsA treatments (including dosing and duration)  Concurrent medications  Weight (kg)  Height (cm)  Waist circumference (cm)  Erythrocyte sedimentation rate (ESR)  C-reactive protein (CRP)  TJC (68 joints)  SJC (66 joints)  Disease Activity index for Psoriatic Arthritis (DAPSA)  Leeds Enthesitis Index (LEI)  Number of digits with dactylitis  Inflammatory Back Pain (ASAS criteria)  Ankylosing Spondylitis Disease Activity Score (ASDAS)  Bath Ankylosing Spondylitis Disease Activity Index (BASDAI)  Skin psoriasis (present/absent)  Nail psoriasis (present/absent)  Health Assessment Questionnaire (HAQ)  * Collected only at baseline |
| **Peripheral spondyloarthritis clinical data (individual visit):**  Diagnosis date*  Symptoms onset date*  Clinical features experienced to date:   - Familial history of psoriasis - Skin psoriasis - Nail psoriasis - Synovitis - Enthesitis - Dactylitis - Axial disease - Uveitis - Inflammatory bowel disease   Current pSpA treatment (including dosing and duration)  Past pSpA treatments (including dosing and duration)  Concurrent medications  Weight (kg)  Height (cm)  Waist circumference (cm)  Erythrocyte sedimentation rate (ESR, mm/h)  C-reactive protein (CRP, mg/dL)  HLA-B27 status  TJC (68 joints)  SJC (66 joints)  Leeds Enthesitis Index (LEI)  Number of digits with dactylitis  Inflammatory Back Pain (ASAS criteria)  Ankylosing Spondylitis Disease Activity Score (ASDAS)  Bath Ankylosing Spondylitis Disease Activity Index (BASDAI)  Skin psoriasis (present/absent)  Nail psoriasis (present/absent)  Health Assessment Questionnaire (HAQ)  * Collected only at baseline |
| **Axial spondyloarthritis clinical data (individual visit):**  Diagnosis date*  Symptoms onset date*  Clinical features experienced to date:   - Familial history of psoriasis - Skin psoriasis - Nail psoriasis - Synovitis - Enthesitis - Dactylitis - Axial disease - Uveitis - Inflammatory bowel disease   Current AxSpA treatment (including dosing and duration)  Past AxSpA treatments (including dosing and duration)  Concurrent medications  Weight (kg)  Height (cm)  Waist circumference (cm)  Erythrocyte sedimentation rate (ESR)  C-reactive protein (CRP)  TJC (68 joints)  SJC (66 joints)  Leeds Enthesitis Index (LEI)  Number of digits with dactylitis  Inflammatory Back Pain (ASAS criteria)  Ankylosing Spondylitis Disease Activity Score (ASDAS)  Bath Ankylosing Spondylitis Disease Activity Index (BASDAI)  Skin psoriasis (present/absent)  Nail psoriasis (present/absent)  Health Assessment Questionnaire (HAQ)  * Collected only at baseline |

**Supplementary material S3.** Biological samples accompanying sheet.

**Supplementary material S4.** Biological sample preparation register.


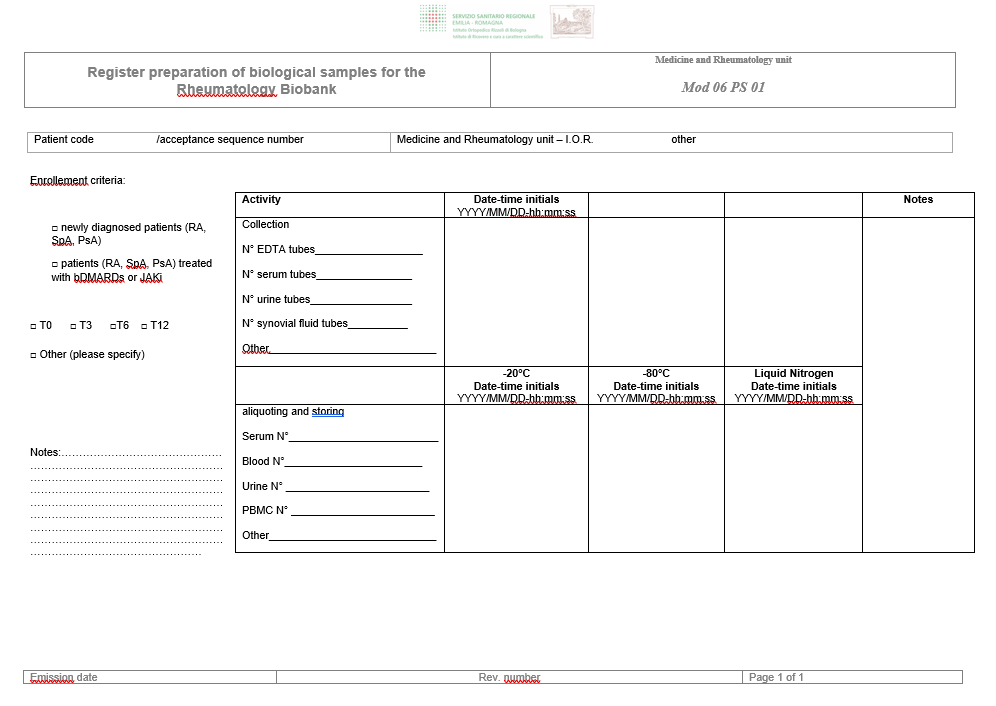

Supplement: Supplementary file 1 [file Data_Sheet_1.docx]
